# Supplementary material for: Drug repurposing for aging research using model organisms
Source: Aging Cell. 2017 Jun 16;16(5):1006–15. doi: 10.1111/acel.12626 (PMC5595691; doi:10.1111/acel.12626)
Supplement: Supplementary file 7 — Data S1 Zip‐Archive of all report cards. [file ACEL-16-1006-s007.zip › RC_ASP.pdf]

ASP

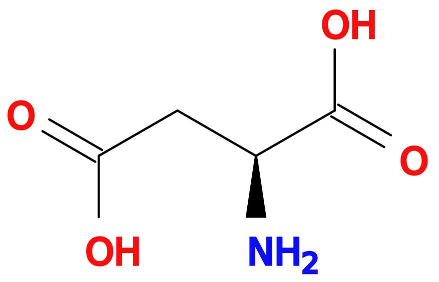

Database identifiers

ChEMBLCompound	CHEMBL274323
DrugBank	DB00128
CHEBI	17053
eMolecules	478712

Ranking

	Rank	Score
Drosophila	328/697	0.472
C. elegans	NA	NA

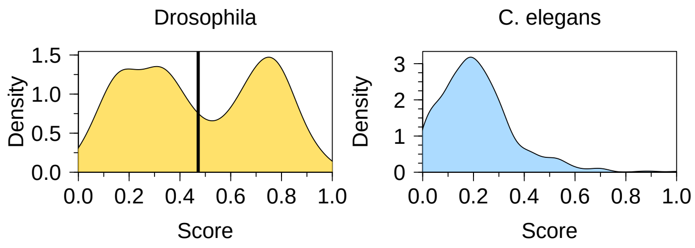

	Ageing implication	Domain conservation	Binding site conservation	Binding affinity	Bioavailability	Lipinski	Promiscuity	Purchasability	Drug approval	**Total**
Drosophila	0.792	0.918	1.0	0.454	(0.9)	0.0	-0.0	0.1	0.075	0.472
C. elegans	NA	NA	NA	NA	NA	NA	NA	NA	NA	NA

Names

- **L-aspartic acid**
- (S)-2-aminobutanedioic acid
- (S)-2-aminosuccinic acid
- Asp
- D
- L-Asparaginsaeure

Roles

neurotransmitter

Status

Approved drug (according to ChEMBL)	No
Classification (according to DrugBank)	approved, nutraceutical
Number of Rule of 5 violations	0
Binding affinity to original target in log units (RF-Score prediction)	4.82
Burns *C. elegans* bioavailability prediction	-4.29

Medical Information

Indicatation: There is no support for the claim that aspartates are exercise performance enhancers, i.e. ergogenic aids.
Mechanism of action: There are also claims that L-aspartate has ergogenic effects, that it enhances performance in both prolonged exercise and short intensive exercise. It is hypothesized that L-aspartate, especially the potassium magnesium aspartate salt, spares stores of muscle glycogen and/or promotes a faster rate of glycogen resynthesis during exercise. It has also been hypothesized that L-aspartate can enhance short intensive exercise by serving as a substrate for energy production in the Krebs cycle and for stimulating the purine nucleotide cycle.
Toxicity: Mild gastrointestinal side effects including diarrhea. LD50 (rat) > 5,000 mg/kg.
Retail: Spectrum Pharmaceuticals
L-aspartic acid powder - 2.46 USD / g

(Information from DrugBank)

Compound Target Characteristics

Argininosuccinate synthase

Best gene implication in ageing for this target family came from gene P09034 annotated in UniProt release 2014_02. Annotation GO 7568 (aging) was Inferred from Expression Pattern

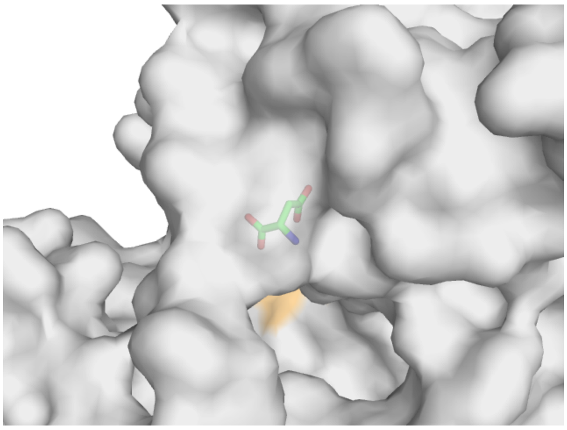

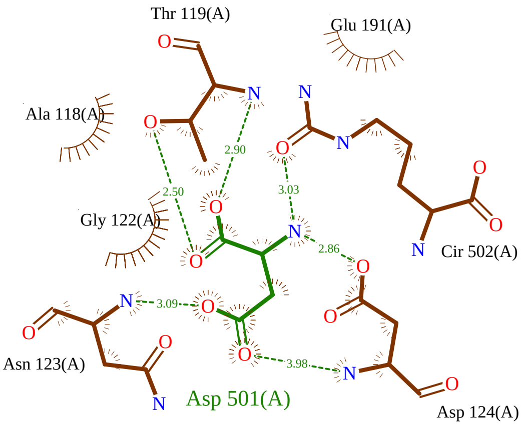

protein	amino acids contacts (binding site)
PDB:2nz2:chainA:P00966	A T G N D E
tr:Q5T6L6:Q5T6L6_HUMAN	A T G N D E
tr:Q5T6L4:Q5T6L4_HUMAN	A T G N D E
sp:P00966:ASSY_HUMAN	A T G N D E
sp:P09034:ASSY_RAT	A T G N D E
sp:P16460:ASSY_MOUSE	A T G N D E
tr:Q3UJ34:Q3UJ34_MOUSE	A T G N D E
tr:J3QNG0:J3QNG0_MOUSE	A T G N D E
sp:O97069:ASSY_DROME	A T G N D E
sp:P22768:ASSY_YEAST	C T G N D E

	whole protein		domain-based		contact-based	
protein	ident	simil	ident	simil	ident	simil
PDB:2nz2:chainA:P00966	1.0	1.0	1.0	1.0	1.0	1.0
tr:Q5T6L6:Q5T6L6_HUMAN	0.48	0.48	0.53	0.53	1.0	1.0
tr:Q5T6L4:Q5T6L4_HUMAN	1.0	1.0	1.0	1.0	1.0	1.0
sp:P00966:ASSY_HUMAN	1.0	1.0	1.0	1.0	1.0	1.0
sp:P09034:ASSY_RAT	0.98	0.99	0.98	0.99	1.0	1.0
sp:P16460:ASSY_MOUSE	0.97	0.99	0.97	0.99	1.0	1.0
tr:Q3UJ34:Q3UJ34_MOUSE	0.97	0.99	0.97	0.99	1.0	1.0
tr:J3QNG0:J3QNG0_MOUSE	0.97	0.99	0.97	0.99	1.0	1.0
sp:O97069:ASSY_DROME	0.53	0.83	0.55	0.84	1.0	1.0
sp:P22768:ASSY_YEAST	0.49	0.81	0.51	0.83	0.83	0.7

CG1315 (UniProt:O97069) annotation

Pathway: Amino-acid biosynthesis; L-arginine biosynthesis; L- arginine from L-ornithine and carbamoyl phosphate: step 2/3.

Pathway: Nitrogen metabolism; urea cycle; (N(omega)-L- arginino)succinate from L-aspartate and L-citrulline: step 1/1.

Subunit: Homotetramer.

(Information from UniProt)
